# Analysis of the operating conditions for UAV-based on-board antenna radiation pattern measurement systems

**DOI:** 10.1371/journal.pone.0245004

**Published:** 2021-02-03

**Authors:** José Núñez, Pedro Orgeira-Crespo, Carlos Ulloa, Inés García-Tuñón

**Affiliations:** 1 Defense University Center, Spanish Naval Academy, Pontevedra, Spain; 2 Department of Mechanical Engineering, Heat Engines and Machines, and Fluids, Aerospace Area, Aerospace Engineering School, University of Vigo, Vigo, Spain; 3 Department of Signal Theory and Communications, University of Vigo, Vigo, Spain; University of Scranton, UNITED STATES

## Abstract

Communications, navigation, and other related systems need to have a well-defined antenna radiation pattern. In onboard vessel systems, the radiation pattern can be much different than the one obtained for an isolated antenna (because of the vessel’s structure and other nearby radiating systems interference). Finding out the onboard antenna’s radiation pattern is a well-known problem for any shipbuilder/owner. The conventional method consists of measuring radiation patterns from a fixed antenna on the coast while the ship is navigating in circles. Recent electronic systems in the market now allow for an alternative method: keeping the ship static while an unmanned aerial vehicle (UAV) circles it, measuring the antenna’s transmitted power. This research paper examines the airspace volume and optimal flight path of an off-the-shelf UAV system for measuring the onboard antenna’s radiation pattern in the presence of physical constraints such as the vessel’s dimensions, safety zone, distance to base, Fresnel’s and multipath distances, and considering the loss due to polarization decoupling between the antenna under test and UAV’s antenna.

## Introduction

Antenna’s characterization implies the determination of certain parameters that affect its behavior, such as directivity, gain, polarization, bandwidth, and radiation pattern [1]. Parameters obtained from onboard radiation pattern studies may differ from theoretical isolated ones, and therefore the system’s performance they serve is commonly degraded. It is of the essence to obtain the antenna’s radiation pattern at its actual location on the ship’s structure and, when possible, having the ship in its own environment (the sea).

**Fig 1:**
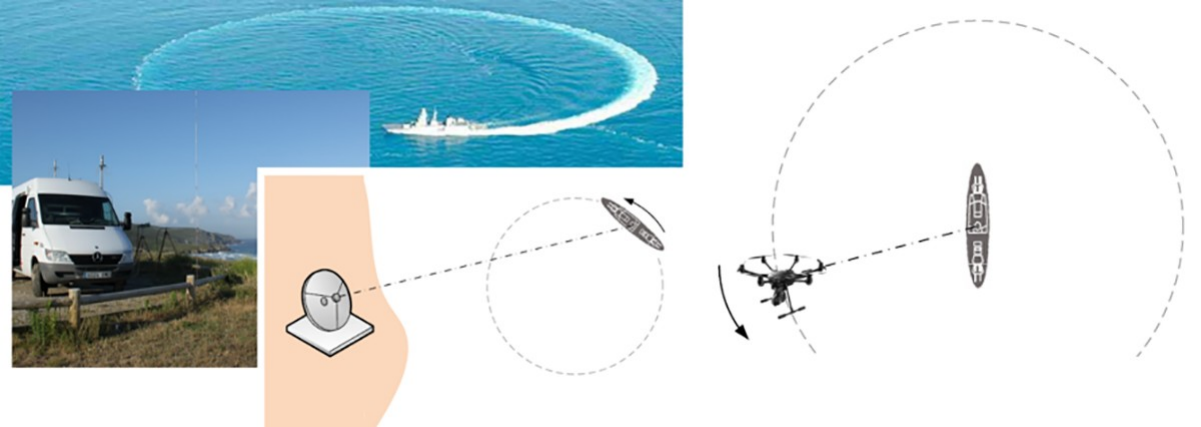
Antenna’s radiation pattern obtention methods: Conventional (left, the ship circles and a probe antenna stays in the coast) and alternative (right, the vessel is static at sea and a UAV circles it).

On-board antenna’s characterization is conventionally performed in the following way (Fig 1): antenna under test begins transmission while the ship sails describing circles, and a second antenna (probe) logs received power values from a fixed point in the coast.

A different approach is feasible as described in [2,3]: the ship keeps static at sea emitting with its test antenna, while the UAV flies in circles with respect to the vessel, measuring received power using its probe antenna. Unmanned vehicles have already been used to obtain radiation patterns, coverage maps, and dangerous radiation maps [4–7]. UAV are included in 2006’s ITU-R SM.2056-1 recommendation for broadcasting antenna’s radiation pattern verification [8]. Although this recommendation is potentially valid for any kind of aircraft, it is clearly focused on airplanes and helicopters, which are the traditional type of aircrafts used in this area. Moreover, the recommendation focuses on terrestrial VHF-UHF antennas and does not state any guideline for other generic types.

This article presents the theoretical study, a synthesis of the simulations carried out in order to confirm the validity of the results obtained and, finally, a case study as an example application of the developed methodology that again serves as a means of validating the proposed method. The novelty of this research lies on the definition of the constraints for the flight mission of a COTS (commercial off the shelf) UAV for the characterization of static vessel’s on-board antennas.

In order to evaluate the performance of ship’s antenna using an UAV describing a circled path flight over the sea, the following limitations must be considered:

- Physical constraints:
  ○ Ship’s dimensions
  ○ Safety distance from the ship
  ○ UAV legislation
  ○ UAV stability
  ○ UAV optimal path due to its limited battery life
- Electromagnetic constraints:
  ○ Fresnel’s distance
  ○ Multipath distance
  ○ Potential signal loss because of antenna’s polarization decoupling

The rest of the paper is structured as follows: in section two, the physical foundations of antenna’s radiation pattern characterization are analyzed. The parameters that affect UAV’s flight for this purpose are taken care of, to define distance restrictions for common transmission bands- those restrictions come mainly from physical and electromagnetic constraints. The effect of the flight mission is also analyzed, determining the consequences on polarization decoupling losses to find a compromise proposal. Section three applies the developed methodology to a particular case of Spanish Navy’s F100 class frigates, defining the flight mission to perform the characterization of the ship’s antenna.

## Materials and methods

### Antenna’s radiation pattern measurement by means of a UAV

Among the methods described in [2,3], in the fixed line of sight scenario, the antenna under test (AUT) and its solidary reference system rotate with respect its own vertical axe. This way, when the AUT is transmitting, reception radiation pattern might be obtained by continuously recording the power values received by the probe antenna (PA). In the proposed methodology, the opposite idea was used–the onboard AUT will be on the ship, static in the sea, whereas the PA will be installed on the UAV in the reception role.

Radiation pattern according to the angular direction in space (spherical coordinates) is described as [1]:

$$F(\theta ,\varphi )=\frac{g(\theta ,\varphi )}{g_{max}}$$ (1)

where *g*(*θ*,*φ*) is antenna’s gain in any direction and *g*_*max*_ the maximum gain.

Friis Eq (1) obtains received power in an antenna *p*_*r*_(*θ*,*φ*,*r*) with the one transmitted (*p*_*t*_) at a certain distance *r* using transmitted (*g*_*t*_) and received (*g*_*r*_) gains for a specific wavelength *λ*:

$$p_{r}(\theta ,\varphi ,r)={\left(\frac{\lambda }{4\pi r}\right)}^{2}p_{t}∙g_{t}(\theta ,\varphi )∙g_{r}(\theta ,\varphi )$$ (2)

If either an omnidirectional antenna (with constant gain in any direction) or a directional antenna with its main lobe keeping pointed towards the AUT is used in reception, the gain can be considered a constant.

Moreover, when the PA antenna describes a circle of radius *R* around the AUT antenna, their relative separation is constant. Since the transmitted power, frequency, and distance are constants when received power *p*_*r*_ is obtained by the PA, AUT’s gain in the AUT-PA direction is obtained as follows:

$$g_{t}(\theta ,\varphi )=K∙p_{r}(\theta ,\varphi ,R)$$ (3)

where the *K* constant that includes the receiving antenna gain, transmitted power, frequency, and distance between antennas and only scales the values in the radiation pattern.

**Fig 2:**
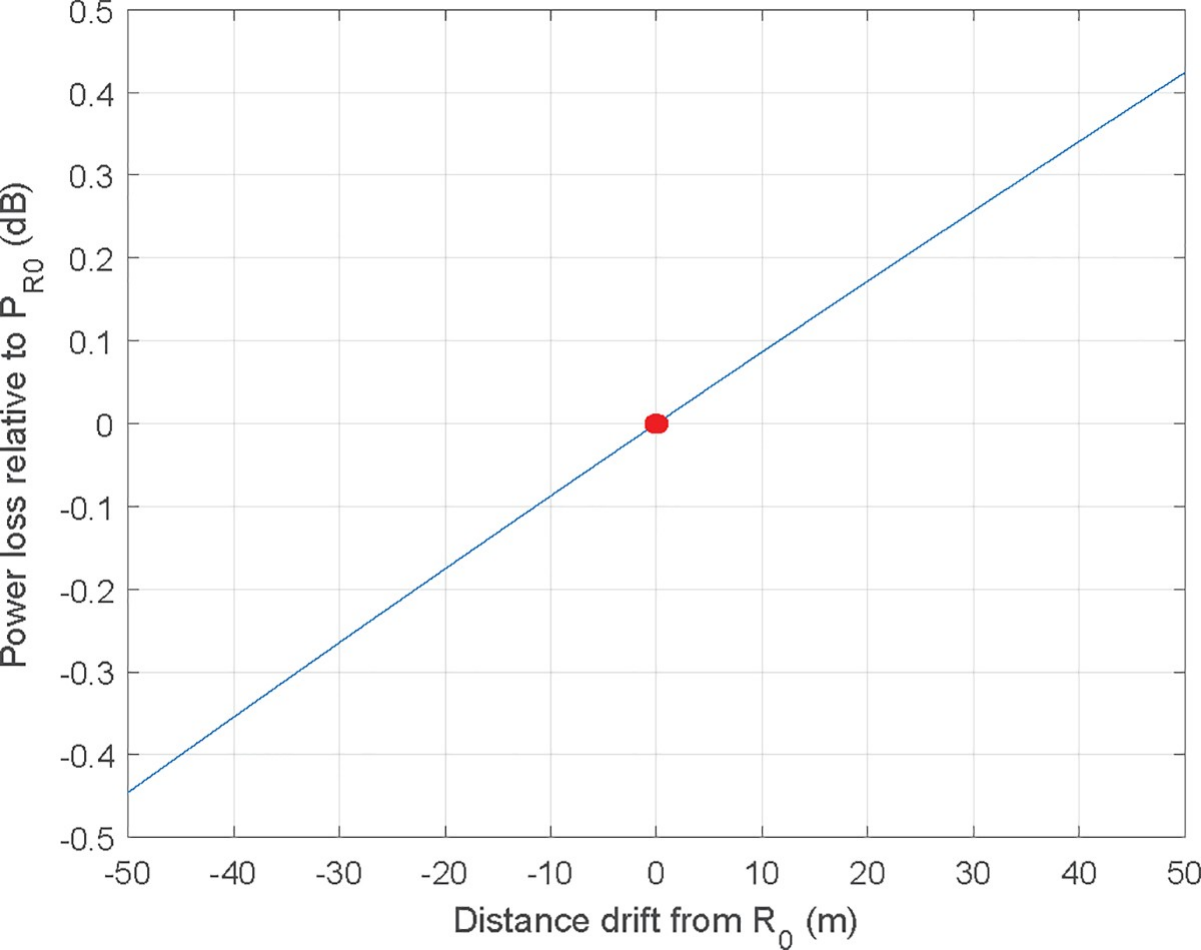
Correction factor that should be applied to the estimated gain if radial distance deviation from the theoretical path exists.

Constant distance is easily achieved by keeping a circle flight path over the static ship with the UAV. In a real measurement environment, constant distance cannot always be ensured during the measurement due to small deviations in the trajectory of the UAV or the ship's own dynamics. In this case, a gain correction based on the distance drift from the theoretical path should be taken into account. This correction, which does not depend on the working frequency, can easily be made from the relative positions of the UAV and the ship during the measurement, obtained from the telemetry data. Fig 2 shows the correction factor that should be applied to the power measurements to compensate the radial distance deviations from the theoretical path between the ship and the UAV.

Usually, the measurement campaigns will be carried out in optimal conditions of little wind and calm sea. In these circumstances, a smooth flight of the UAV can be guaranteed as well as a reduced movement of the ship. In doing so, the flight algorithms will guarantee the correct alignment between the PA and the AUT antenna. However, if the movements of the UAV or the ship are significant, additional losses will occur which must be evaluated.

### Flight volume determination

UAV’s flight for pattern radiation determination must be planned according to the factors mentioned in the introduction. The first step is to determine flight’s volume, determined by flight’s elevation and flight surface (security surface around the ship and correct measuring distance).

#### Far field minimum distance

**Fig 3:**
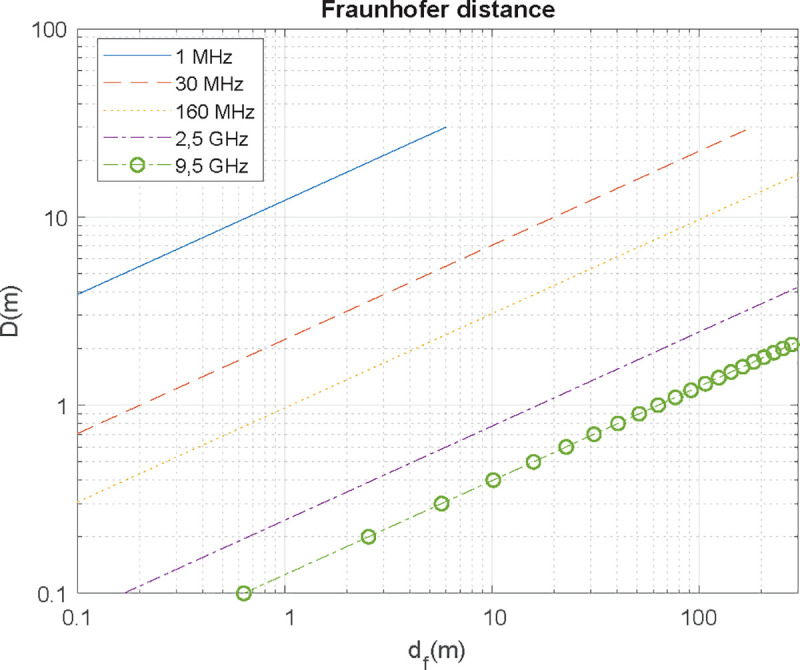
Far field distance as a function of antenna's dimension for different wavelengths.

The Fraunhofer distance *d*_*f*_, that is, the minimum distance to consider the far field conditions [1,9], is the minimum distance from the antenna to measure its radiation pattern that guarantees that the angular distribution is not depending on the distance:

$$d_{f}=\frac{2D^{2}}{\lambda }$$ (4)

where *D* is antenna’s maximum dimension and *d*_*f*_≫*λ*. In Fig 3 antenna’s dimension is plotted against Fraunhofer’s distance. For each antenna (which has an implicit working frequency) a minimum measurement distance must be chosen to ensure that these measurements are made under far-field conditions. For example, an X-band (9.5 GHz) parabolic reflector antenna with a diameter of 1 m must be characterized at a minimum distance of 60 m.

The value of *D* is determined by the size of the antenna chosen based on criteria such as the working frequency and its application. Consequently, the minimum distance according to far field’s criterion is obtained by: first, by determining antenna’s maximum dimension (wire size for monopole-dipole antennas, dish size for satellite antennas, and longest aperture length for horn type ones) and second, using (4) to calculate the Fraunhofer distance. As seen on Fig 3, far field’s distance is only critical for high frequencies in the order of GHz.

#### First Fresnel ellipsoid’s height

**Fig 4:**
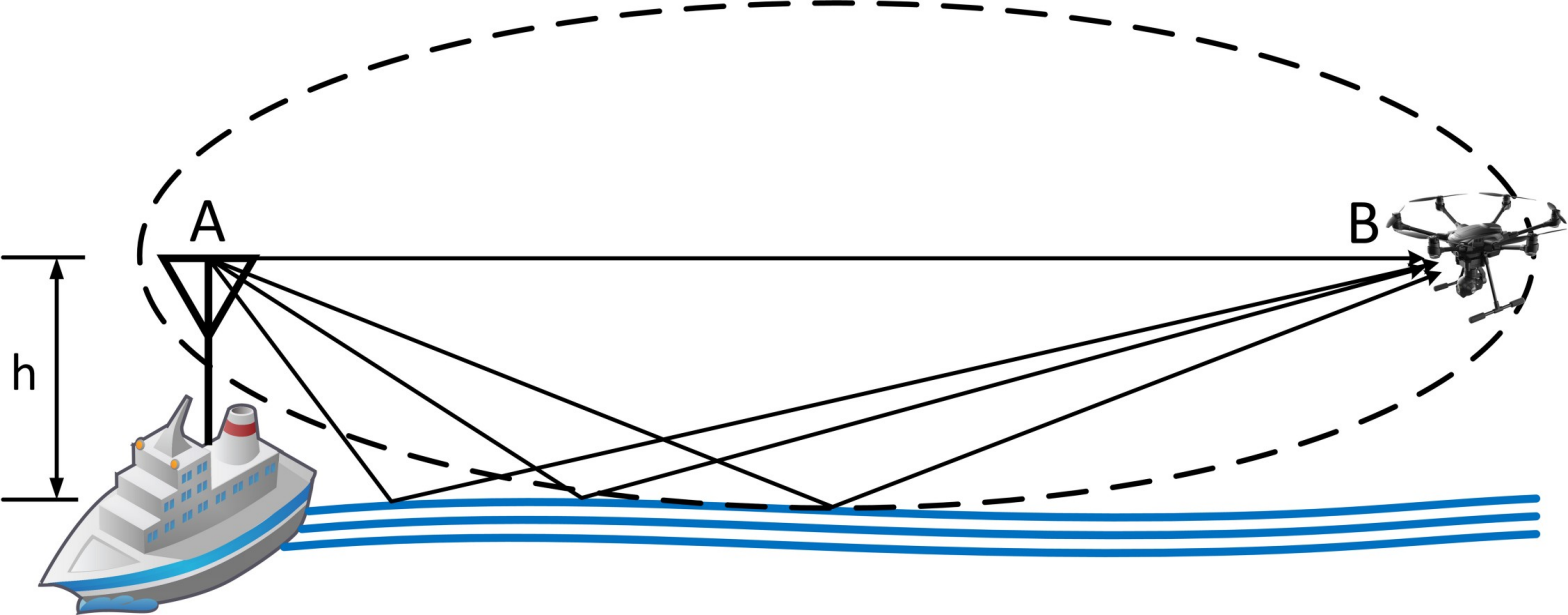
The two possible flight heights of the UAV and their influence on the clearance of the first Fresnel ellipsoid.

The first Fresnel zone [10] must be free of any obstacle and consequently the level flight (*h*) needs to be higher than the first Fresnel zone radius *R*_*1*_. At sea, under controlled maritime flight, the only possible obstacle between the AUT and the PA is sea surface. There are, accordingly, two possible options for flight level as depicted in Fig 4: either same as the AUT’s or higher:

The worst-case according to this criterion is when both antennas are at the same height and the first Fresnel ellipsoid’s height would be:

$$R_{1}=8.66\sqrt{\frac{d}{f}}$$ (5)

where *d* is AUT-PA distance (m) and *f* the frequency (MHz). As Fig 4 shows, this effect is only relevant for low frequencies.

**Fig 5:**
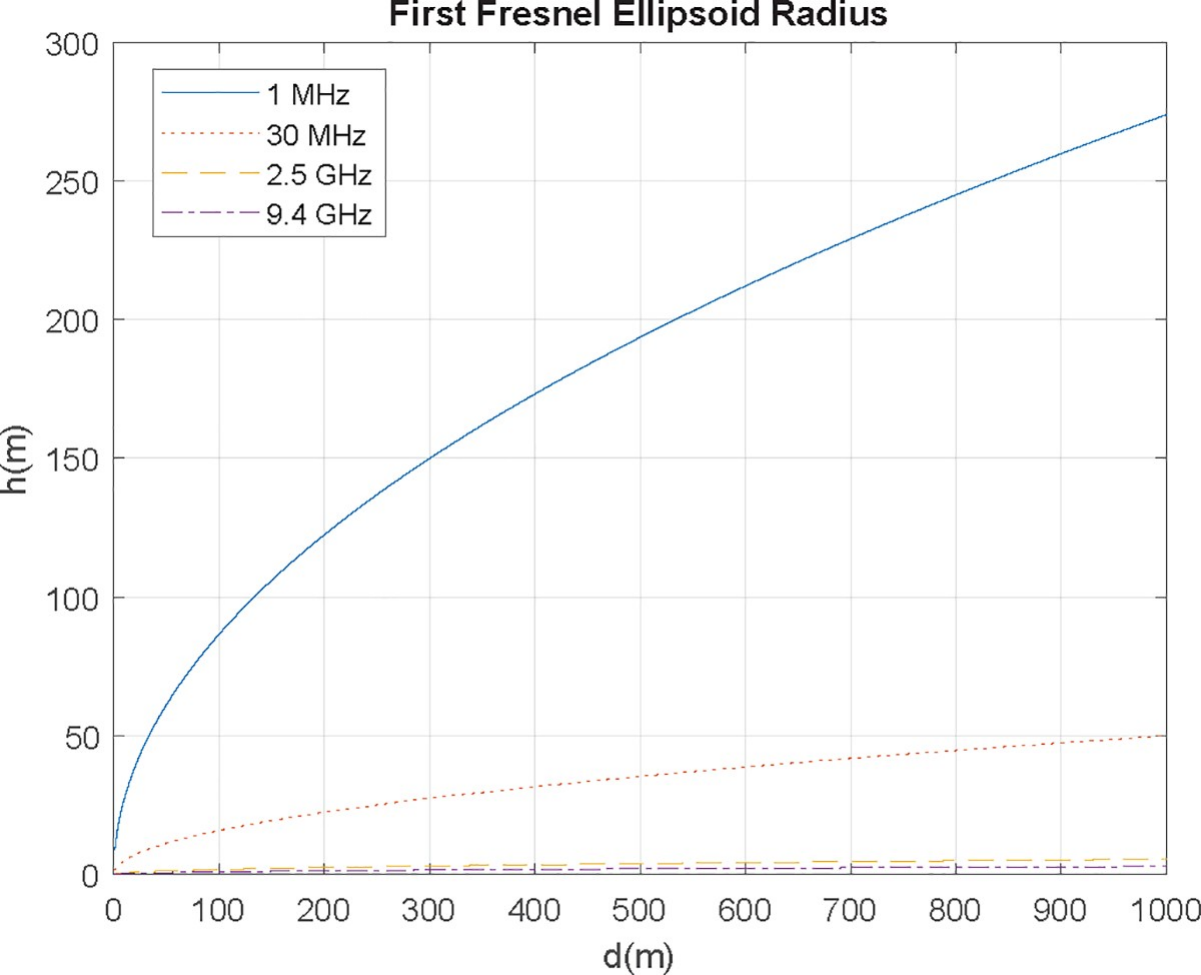
The first Fresnel's ellipsoid radius (flight level) for different frequencies.

The best-case scenario is for a UAV flying over AUT’s height, as depicted in Fig 5, as the ellipsoid is always above sea level.

Since the minimum flight level lowers as the frequency increases, communication antennas in HF, VHF, and UHF bands require a higher flight level than radar SHF antennas. For example, frequencies over 300 MHz need up to 16 m height for distances lower than 1 km; HF band, up to 1 km needs flight level in the 20–160 m range, while VHF ranges 10–50 m.

#### Multipath mitigation distance

**Fig 6:**
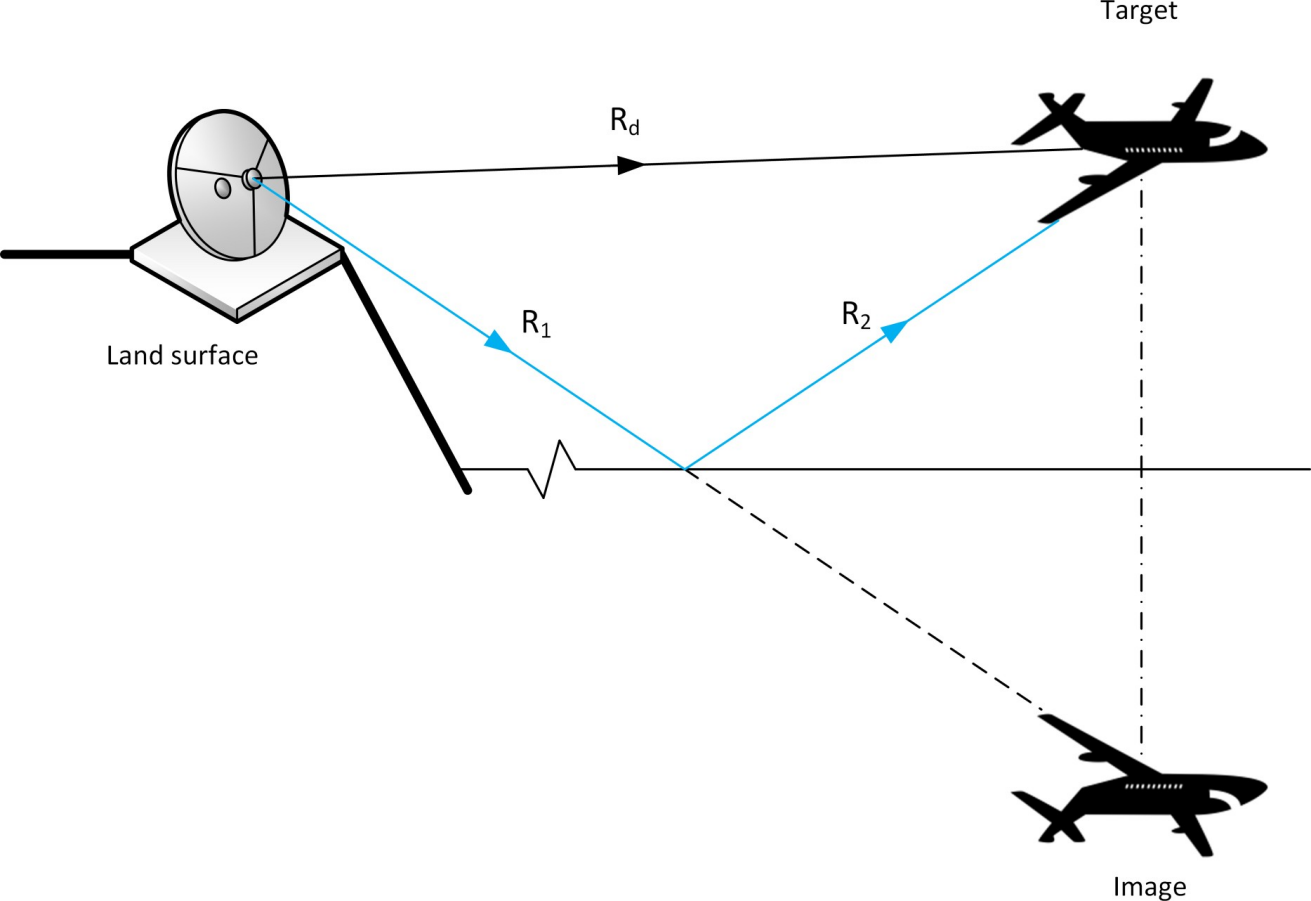
Multipath issue based on the two-ray ground-reflection theory.

Transmission from AUT may reach the UAV through different trajectories because of sea reflections as shown in Fig 6.

**Fig 7:**
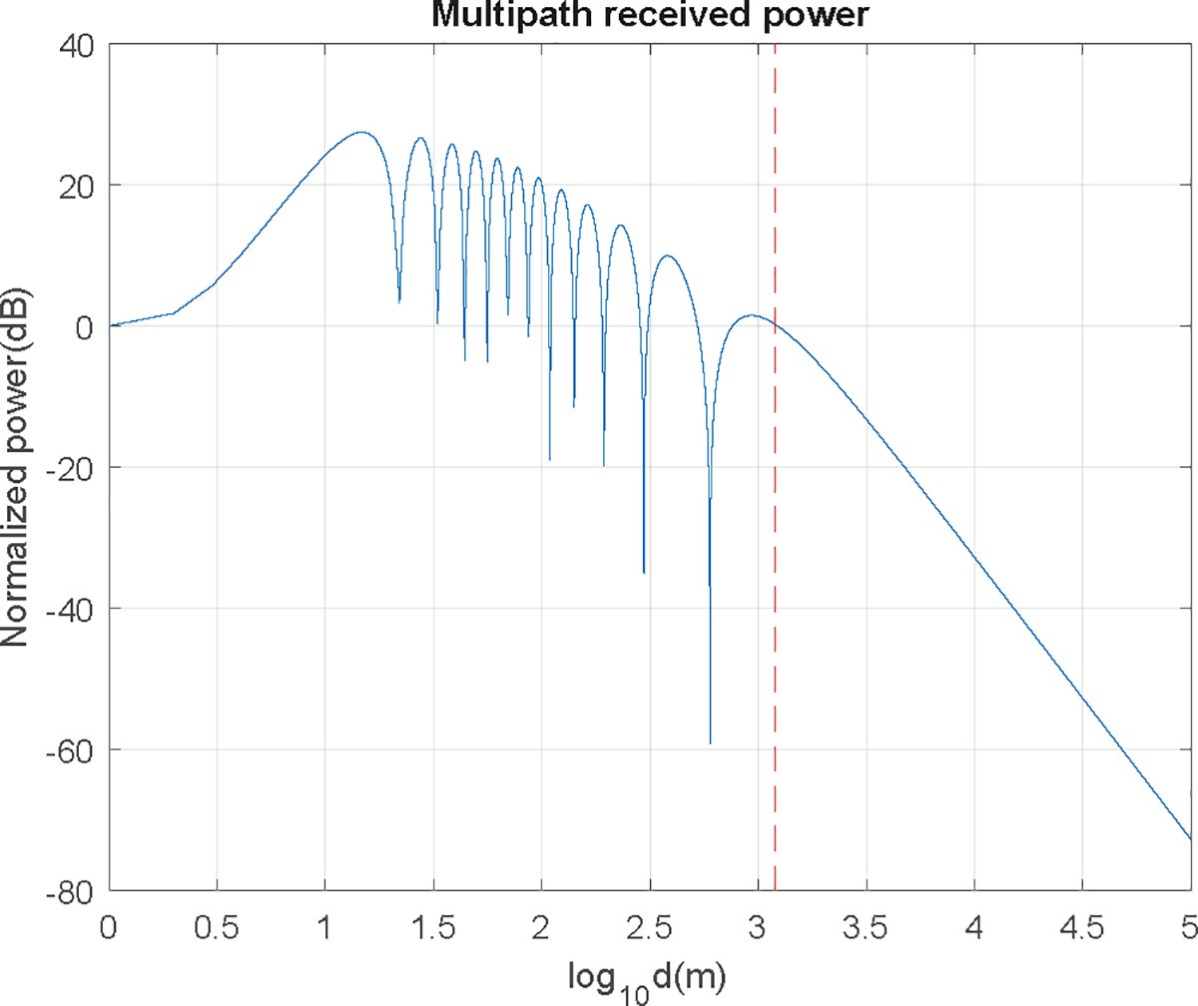
Received power at f = 900 MHz, having h_AUT_ = 2 m and h_UAV_ = 50 m.

One approach to model this interference effect is to use the simple two-ray model [11]. The interference between the two signals considering specular reflection on the flat seawater, i.e., reflection coefficient Γ = −1, has a received signal pattern against distance as exhibited in Fig 7.

The critical distance *d*_*c*_, is the distance after which the signal power falls off proportionally to *d*^−4^ and no lobulation exists (red dotted line in Fig 7). It can be defined as:

$$d_{c}=4\frac{h_{AUT}∙h_{UAV}}{\lambda }$$ (6)

For the case of 900 MHz frequency, flying at 50 m over a 2-meter antenna shown in Fig 7, critical distance is at 1200 m. Over *d*_*c*_ received power is inversely proportional to *d*^4^. For the region where *h*_*UAV*_ *< d < d*_*c*_, power descends inversely proportional to *d*^2^, and propagation in free space can be assumed. The region where *d < h*_*UAV*_ has no interest as the received power can be considered constant. For distances under the critical distance, there is a lobulation pattern, resulting in remarkable power fluctuations. Although radiation pattern obtention should be performed at distances greater than the critical distance from a practical point of view, most of the times this will not be possible, according to (6). For instance: should AUT and UAV be at 10 m high; according to (6), distance should be 40 m for HF, and 4 kilometers for UHF-H (far from UAV’s typical autonomy), so distance will be under critical, with certain restrictions as shown in the next section.

**Fig 8:**
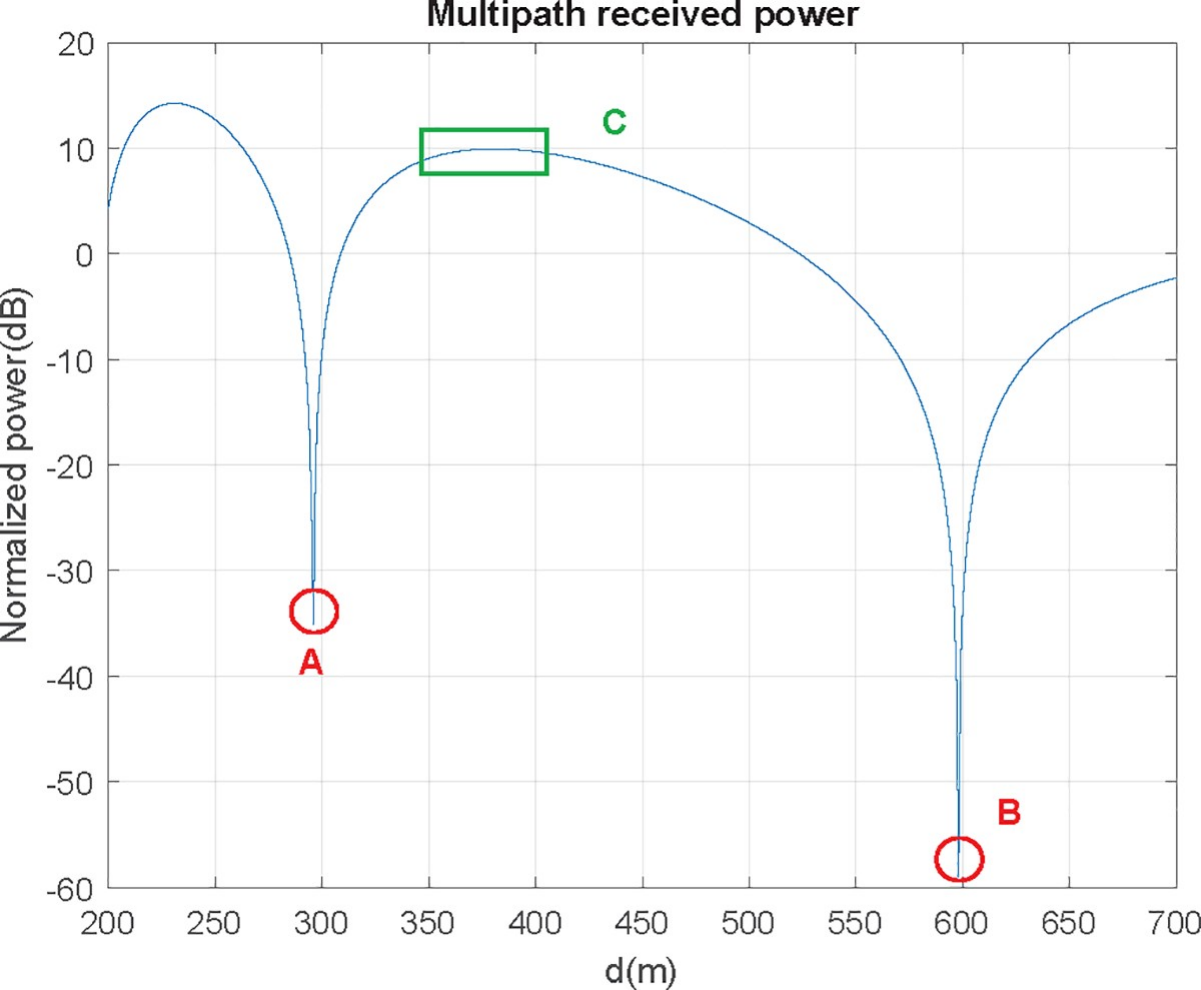
Avoidable and recommended measurement zones due to multipath effect (f = 900 MHz, h_AUT_ = 2 m, h_UAV_ = 50 m).

**Fig 9:**
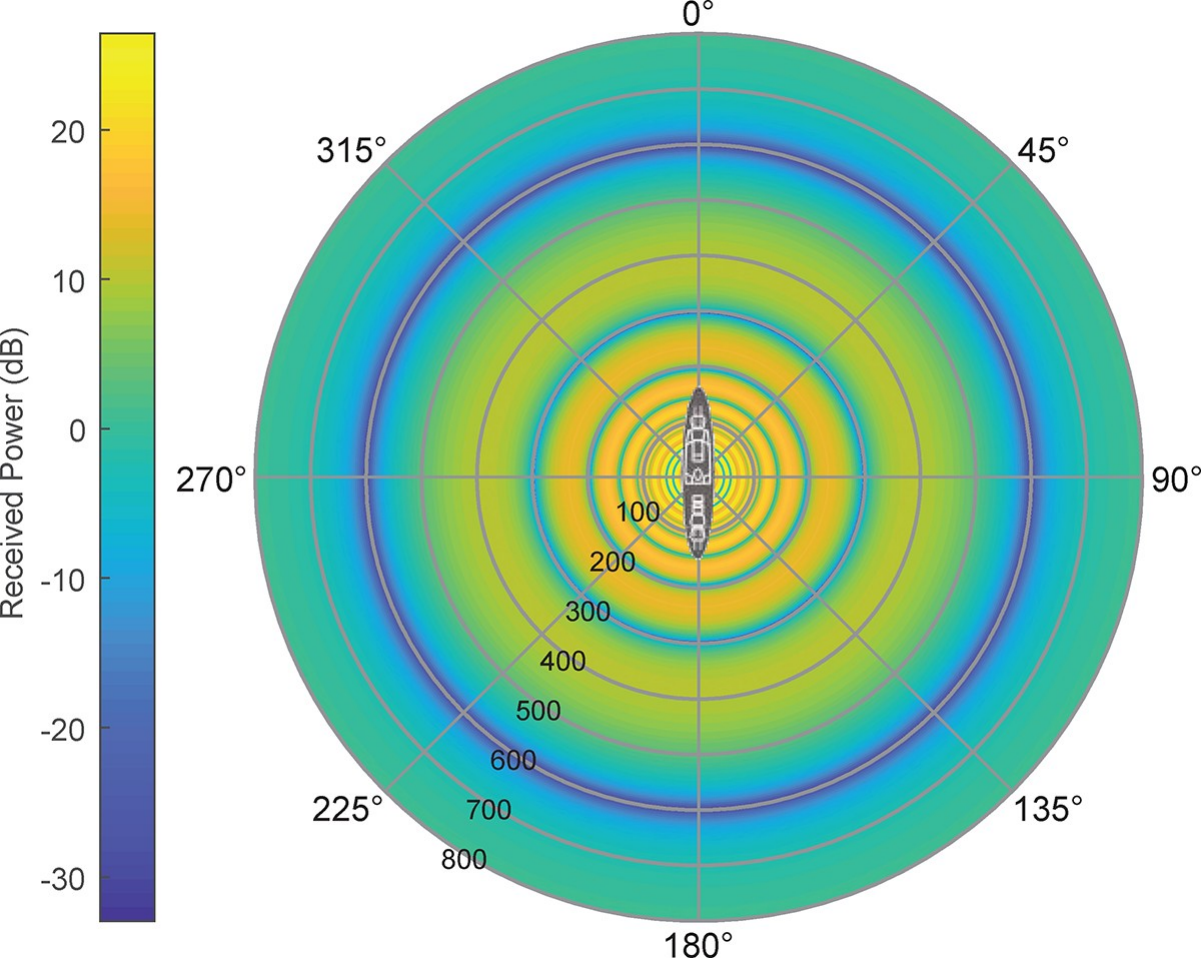
UAV's received signal power in AUT environment (0–800 m range).

**Fig 10:**
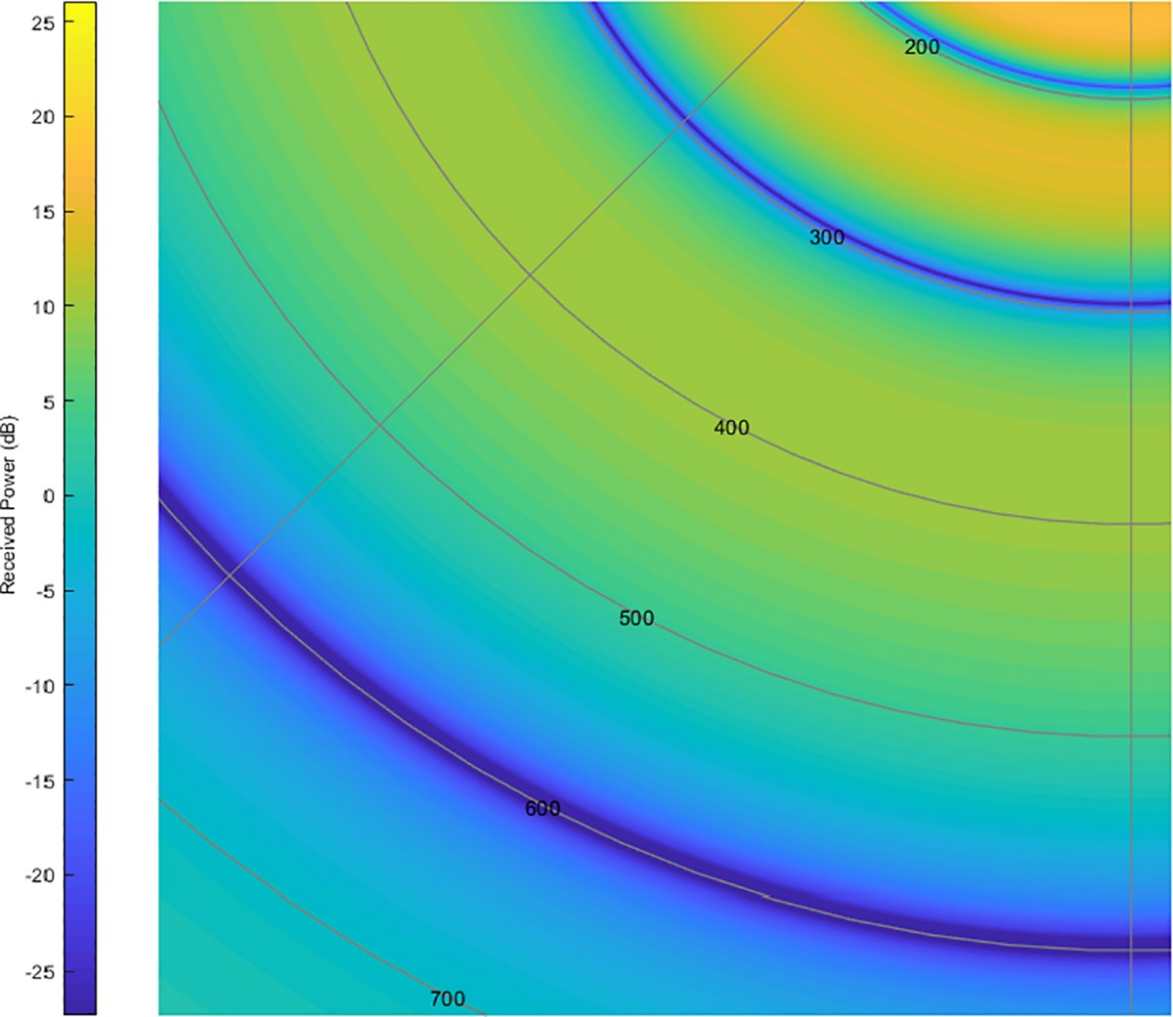
UAV's received signal power in AUT environment (detail on 300–600 m range).

In Fig 8 the focus is in the 200–800 m range, which is clearly under critical distance (1200 m). In this range two severe signal level fading can be found (points A and B): if flight level is within the 300–600 range, received power will be greater than the one obtained in free space and theoretically zero at the extremes. In conclusion: distances where received power nulls should be avoided, and optimum distance is found at an outer ring, where received power is as plain as possible (C zone):

UAV received power reception can be seen in Figs 9 and 10, where the minimum signal rings at 300 and 600 m are shown:

As shown in Figs 9 and 10, in the 350–400 m range, the measuring gradient is 1.5 dB (within measurement uncertainties range). Subsequently, for this case, the UAV should circle its objective at a 375 m distance, having a ±25 m maximum viable discrepancy in order to obtain a measurement error under 1.5 dB according to this criterion.

#### Maximum distance according to optical range

Measurement distances should not exceed radio horizon, according to [12]:

$$d_{h}=3.57\sqrt{\frac{4}{3}h}$$ (7)

where *d*_*h*_ is expressed in kilometers and *h* is the biggest of the heights of the AUT and the UAV in m. This equation considers certain ray bending due to tropospheric diffraction at standard international atmosphere [12] (for a 10 m antenna’s height, the maximum distance according to this criterion would round 13 kilometers).

#### Limitation due to ship’s dimensions

UAV must keep a safety distance with the ship carrying the AUT. Having navy from different countries with different security distances, a distance of 1.5 times the length of the ship was selected. For instance, for a 230 m long ship (LHD Spanish Navy class), the minimum flight radius is 350 m, resulting in a circle path of *L* = 2·*π*·350≈2200 m (plus take off and approach). For a small patrol ship (Segura Spanish Navy class), the radius would be 80 m, resulting in 520 m circle flight. Finally, a safe distance from the ship to coast for this test must also be considered, to be added as an approach path.

#### Legislation restrictions

Radiation pattern measurements for onboard antennas need to be performed at open sea, out of controlled airspace zones. As of writing this paper, drone legislation in Europe is under Commission Delegated Regulation (UE) of 12 March (2019/945). Consequently, there are two choices with regards to remote piloting: VLOS-EVLOS or BVLOS. Visual line of sight (VLOS) and extended visual line of sight (EVLOS) flights are allowed when either the pilot (VLOS) and/or an observer in radio contact with it, are within 500 m range horizontally, at no more than 120 m, or over the tallest obstacle within a 150 m range (from the UAV). Beyond visual line of sight (BVLOS) flights are also permitted within the direct radio range, but only for UAV’s under two kilograms.

### Circle path flight strategies

To achieve the circle path flight described in the previous sections, two strategies were selected:

#### Circular path flight with polygonal approach

The idea behind this method is to split the circular path in straight lines of a polygon inside the circle. As for flight plan, the strategy consists of selecting equidistant points in the circle and command the UAV to follow those points describing a polygon. For any polygon inscribed in a circle with radius R, the length of any of its sides is:

$$L_{n}=2\;R\;sin\left(\frac{\pi }{n}\right)$$ (8)

where *L*_*n*_ = 2 *πR* when n tends to infinity, and total flight equals *L* = *n L*_n_

#### Circular path flight following a trajectory

**Fig 11:**
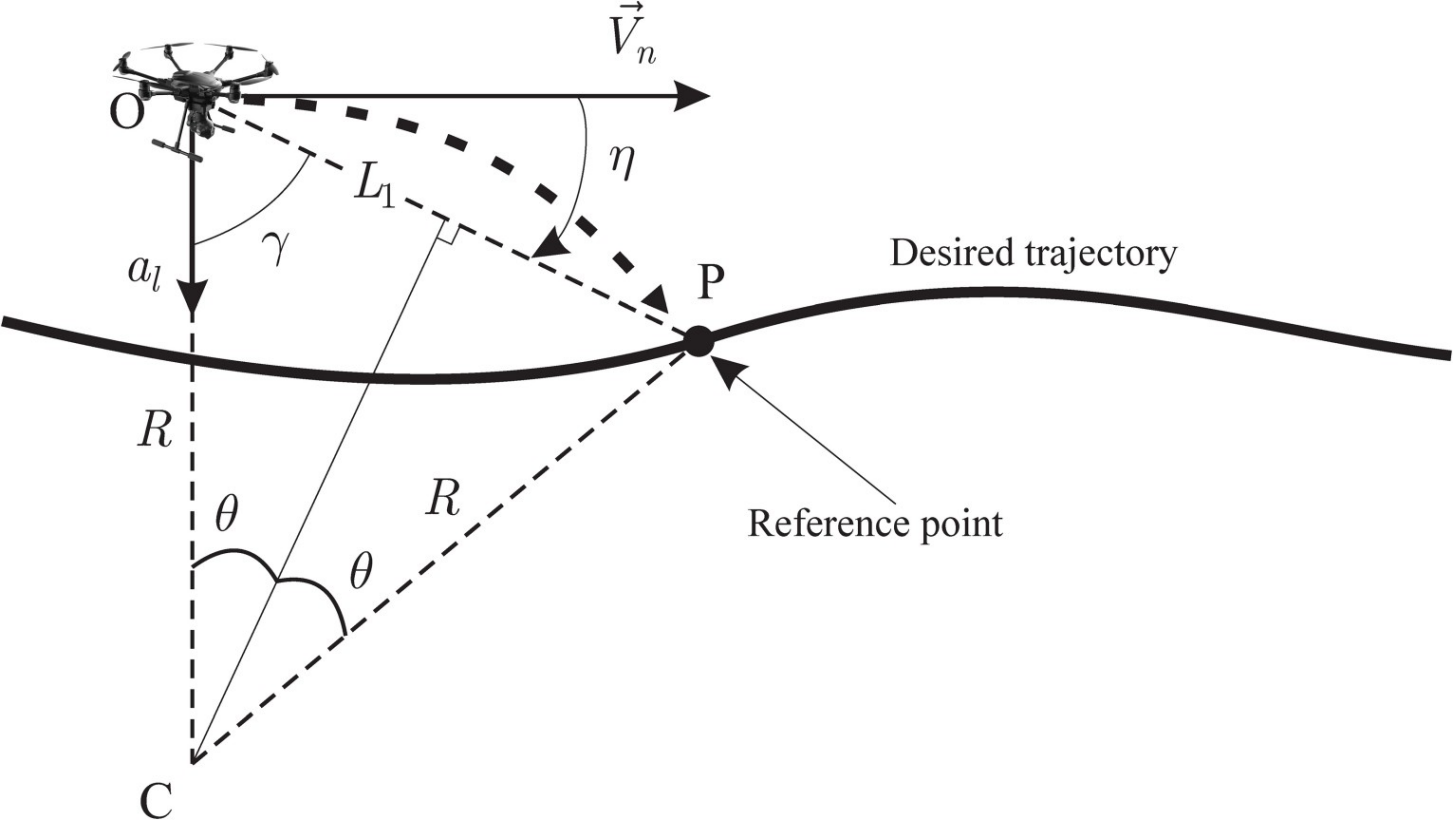
Circular trajectory calculated for lateral guidance.

UAV flight guidance has been studied in [13]. In [14], a proportional guidance algorithm specific to circular flight path is implemented. As shown in Fig 11, for the horizontal flight path [15], given a certain desired flight path, we may draw an arc from UAV’s origin (point O); that arc would intersect desired flight path in a reference point (P). R is the radius of the OP arc, *L*_1_ is the line between O and P, and η is the angle between UAV’s velocity vector $\vec{V_{n}}$ and *L*_1_.

Lateral acceleration needed to take the UAV to P (reference point) following the arc would be:

$$a_{l}=\frac{V_{n}^{2}}{R}=\frac{2V_{n}^{2}}{L_{1}}\sin\;\eta \approx 2\frac{V_{n}}{L_{1}}\left(\dot{y}+\frac{V_{n}}{L_{1}}y\right)$$ (9)

where UAV’s ground speed would be $V_{n}=\sqrt{V_{x}^{2}+V_{y}^{2}}$.

Lateral acceleration makes the UAV change path towards the desired trajectory, changing course. This acceleration should decrease as the error (separation between desired path and actual trajectory) decreases, until they match. Under this control law, acceleration commands generated by navigation system match the centripetal acceleration, resulting in null error for a circular path. Under windy conditions, this guidance algorithm performs better than others [15], since UAV’s ground speed is used to generate acceleration command, intrinsically adapting to inertial speed changes due to wind effect.

#### Polarization decoupling

Eq (2) is Friis’s simpler formulation and it does not include terms representing attenuations found in real radio links. One of the most important is the polarization decoupling factor, l_pol_, defined as the relationship between received power and the one the antenna would receive in maximum polarization coupling circumstances. It would be calculated as:

$$l_{pol}={\left|{\hat{e}}_{inc}(\theta ,\varphi )∙{\hat{e}}_{r}(\theta ,\varphi )\right|}^{2}$$ (10)

Where ${\hat{e}}_{inc}$ is incident field’s polarization unit vector at the receiving antenna and ${\hat{e}}_{r}$ is receiving antenna’s polarization unit vector. To achieve perfect polarization coupling (*l*_*pol*_ *=* 1), the incident wave must have the same polarization as the receiving antenna. If incident’s wave polarization has not changed since it was transmitted, it will match the one of the emitting antenna. In this case, it can be assumed that polarization will not change considering wave’s trip (short), and the test is performed under no circumstance where polarization varies (objects in wave’s path, rain, snow, fog, etc.)

**Fig 12:**
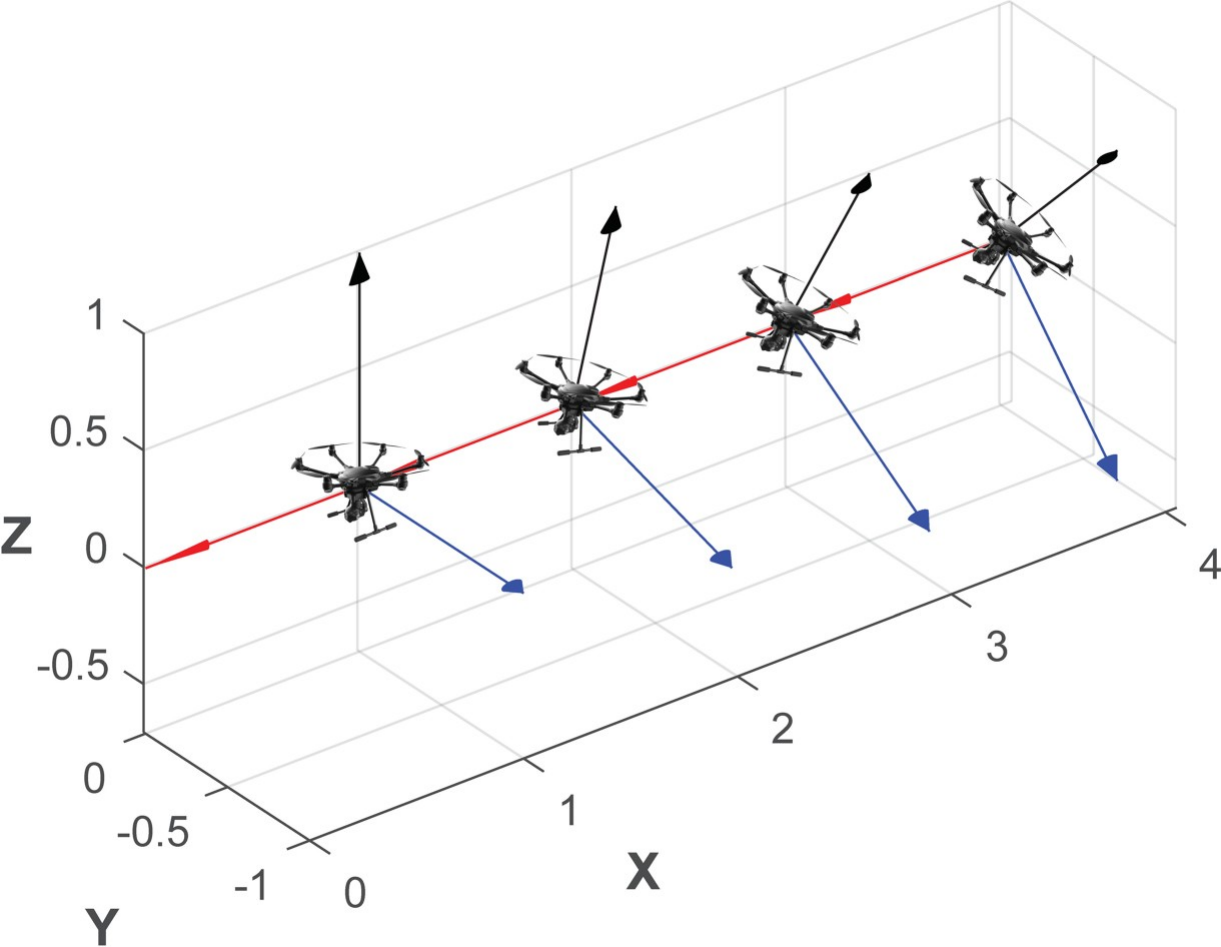
Polarization vector evolution at receiving antenna when a UAV is rolling (rotating with respect to the x axis).

**Fig 13:**
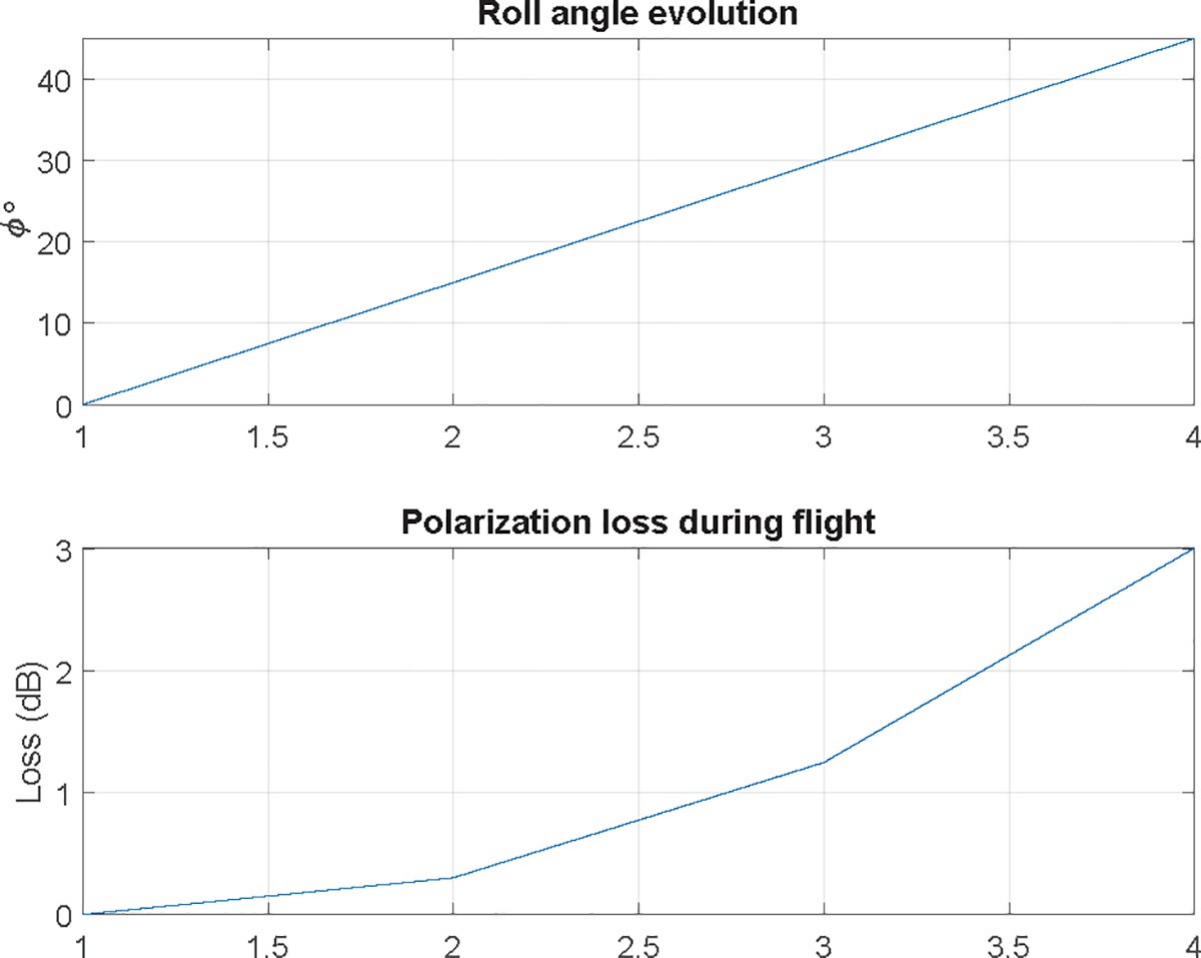
Roll angle evolution and decoupling losses due to rotation with respect to the x axis.

Let us assume now that the transmitter antenna is configured using linear vertical polarization in coordinates origin, aligned with *z* axis; receiving antenna on the UAV is also vertically polarized, aligned with its own *z* axis. Using NED coordinate system (North-x, East-y, Down-z) for the UAV, its yaw (rotation with respect *z*) will not change the polarization match between the transmitting and receiving antennas, but the pitch and roll will change. Consequently, when receiving antenna is boarded on a UAV, its polarization vector is in motion, fixed to UAV’s coordinate system. Although the flight control algorithms will ensure that pitch and roll movements are as low as possible and the receiving antenna is always pointed towards the transmitting antenna, they will generate a change in receiving antenna polarization. As the receiving antenna rotates with respect the *x* axis, while moving along it, the motion and loss due to rotation are similar to the ones depicted in Figs 12 and 13.

**Fig 14:**
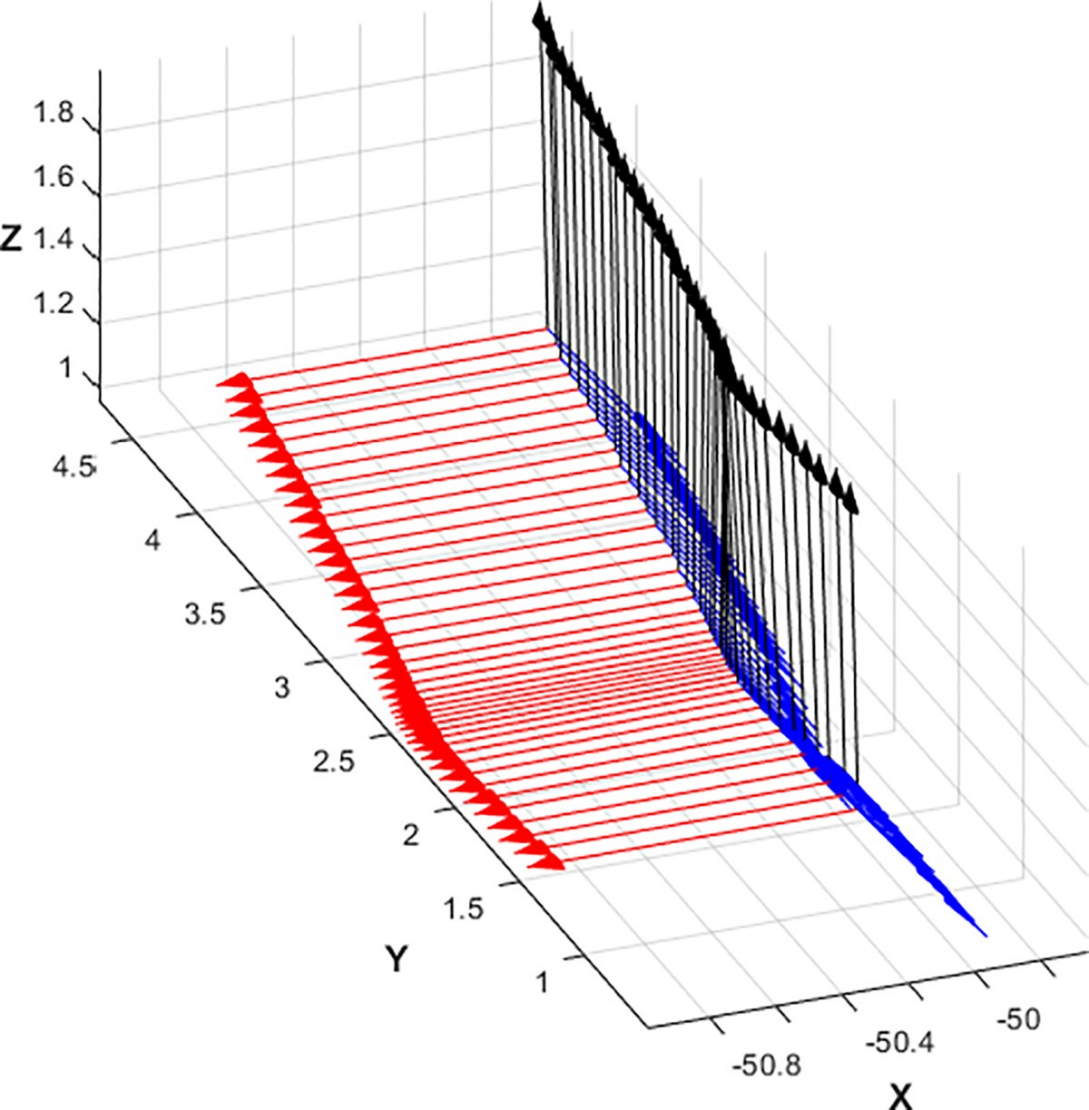
Polarization vector evolution at receiving antenna in a real flight.

**Fig 15:**
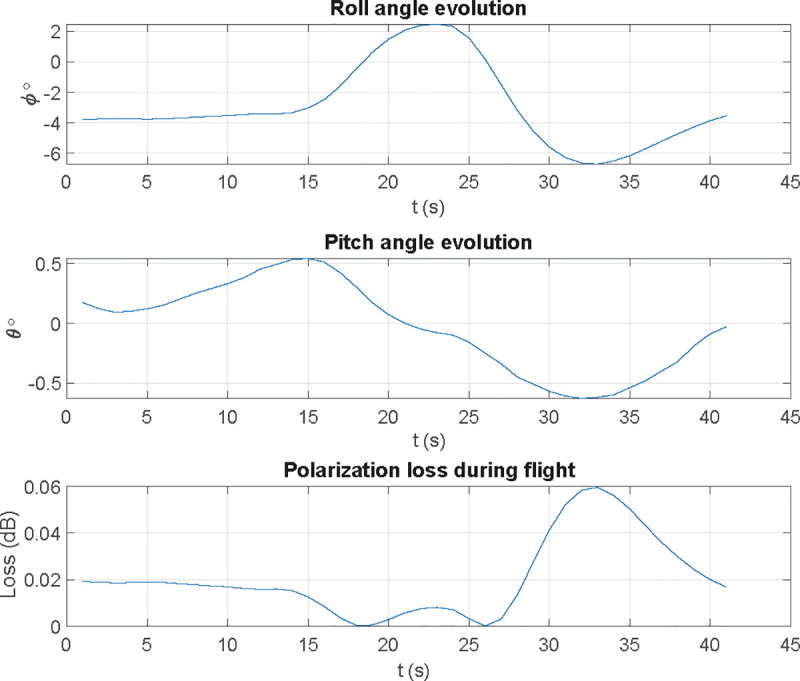
Decoupling losses in a real flight for pitch and roll motions.

In a real flight, nevertheless, once we determine attitude angles, flight angle evolution and decoupling losses are show in Figs 14 and 15:

## Results and discussions

### Flight planning for F-100 frigates on-board antenna’s test

Previous sections described the boundary conditions to set a flight plan for the onboard antenna’s radiation pattern analysis using a UAV. Flight envelope is defined by its maximum and minimum radius (R_max_, R_min_), and its maximum and minimum flight level (h_max_, h_min_)

The Spanish Navy F-100 class frigates were the test case to validate this technique. The parameters for this type of ships can be found Table 1:

**Table 1:** F100 class main dimensions and equipment.

Parameter	min	max
Overall length	146.7 m	
Beam	18.6 m	
Antenna’s base height	21.5 m	
HF communications	1.5 MHz	30 MHz
Link-16 | Link-22	1.5–30 MHz	225–1215 MHz
Inmarsat	1500 MHz	1600 MHz
Secondary radar IFF (CIT-25D)	1090 MHz	
Navigation radar (AN/SPS-67)	5450 MHz	5825 MHz
Satcom (TNX-100V2)	7.25 GHz	7.9 GHz
Navigation radar (ARIES)	8 GHz	12.5 GHz

Using the criteria shown in previous sections, we obtain Fraunhofer’s distance (*d*_*f*_), critical distance (*d*_*c*_), and distance due to ship’s dimensions (*d*_*tb*_). As discussed in section 2.2.3, measurement distance should be over critical distance to avoid field lobulations for being in the *h*_*UAV*_ *< d < d*_*c*_ zone, as show in Table 2.

**Table 2:** Minimum flight distance according to criteria.

	*d*_*f*_ (m)	*d*_*c*_ (m)	*d*_*tb*_ (m)
Coms. HF (1.5–30 MHz)	1.5	10–185	220
Coms. VHF-UHF (30–3000 MHz)	30	185–18500	
Coms. & radar SHF (3–12 GHz)	200	18500–73960	

As key conclusion, we may infer that in VHF low’s band, measurement can be taken over critical distance, since that distance is within average UAV’s range. On the other hand, in the rest of the VHF band and in UHF-SHF, it is impossible not to be affected by multipath and a second iteration is necessary to obtain the right distance. Since minimum distance due to ship’s dimensions is greater than Fraunhofer, *d*_*tb*_ will be used as minimum value. Maximum value is limited by legislation constraints to 500 m as shown in section 2.2.6, so Table 2 evolves to Table 3:

**Table 3:** Minimum flight distance according to criteria (reduced version).

	*d*_*min*_ (m)	*d*_*c*_ (m)	*d*_*max*_ (m)
Coms. HF (1.5–30 MHz)	220	-	500
Coms. VHF (30–300 MHz)		185–1850	
Coms. UHF (300–3000 MHz)		1850–18500	
Coms. & radar SHF (3–12 GHz)		18500–73960	

**Fig 16:**
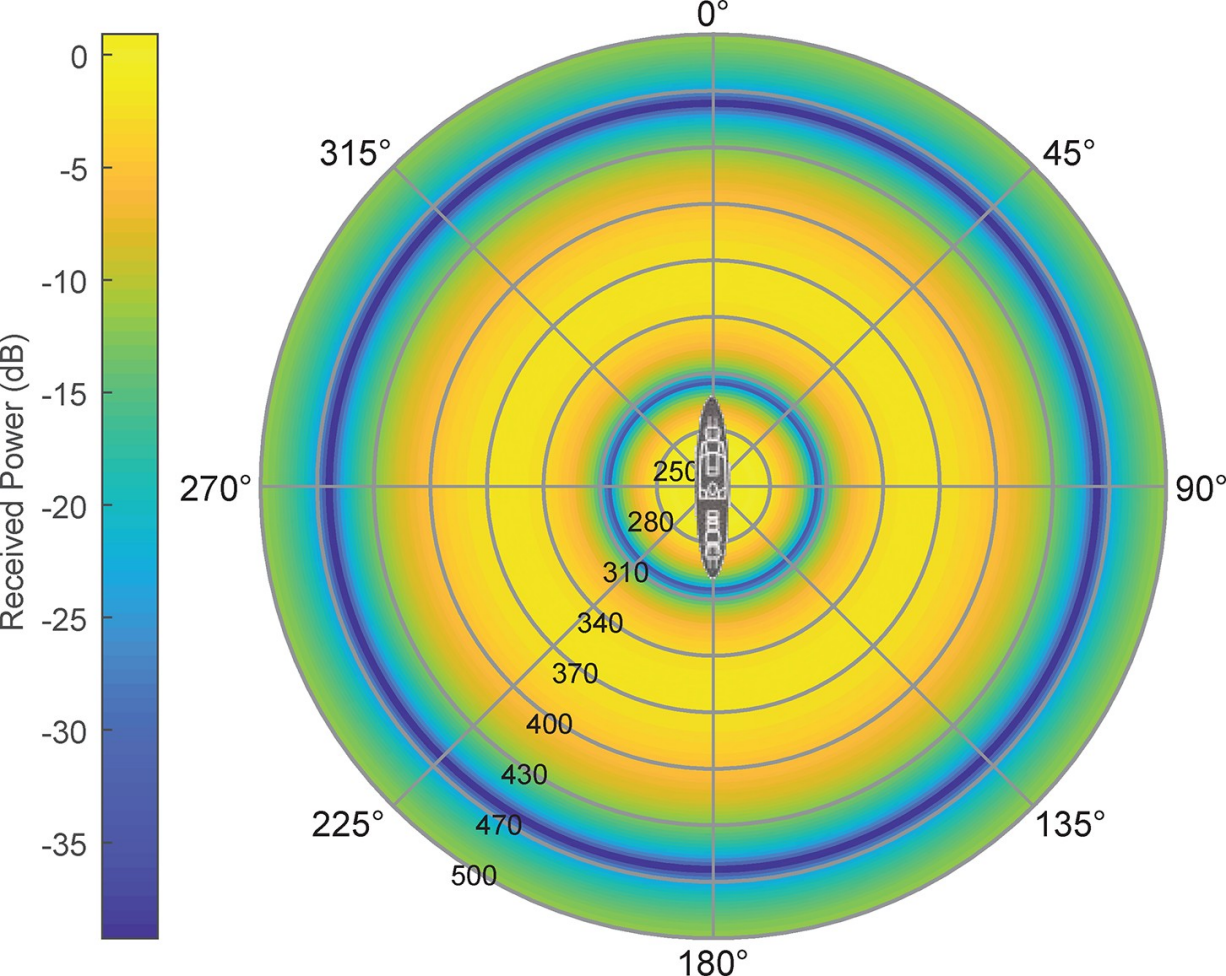
Multipath scenario for f = 300 MHz, h_UAV_ = h_AUT_ = 21.5 m.

**Fig 17:**
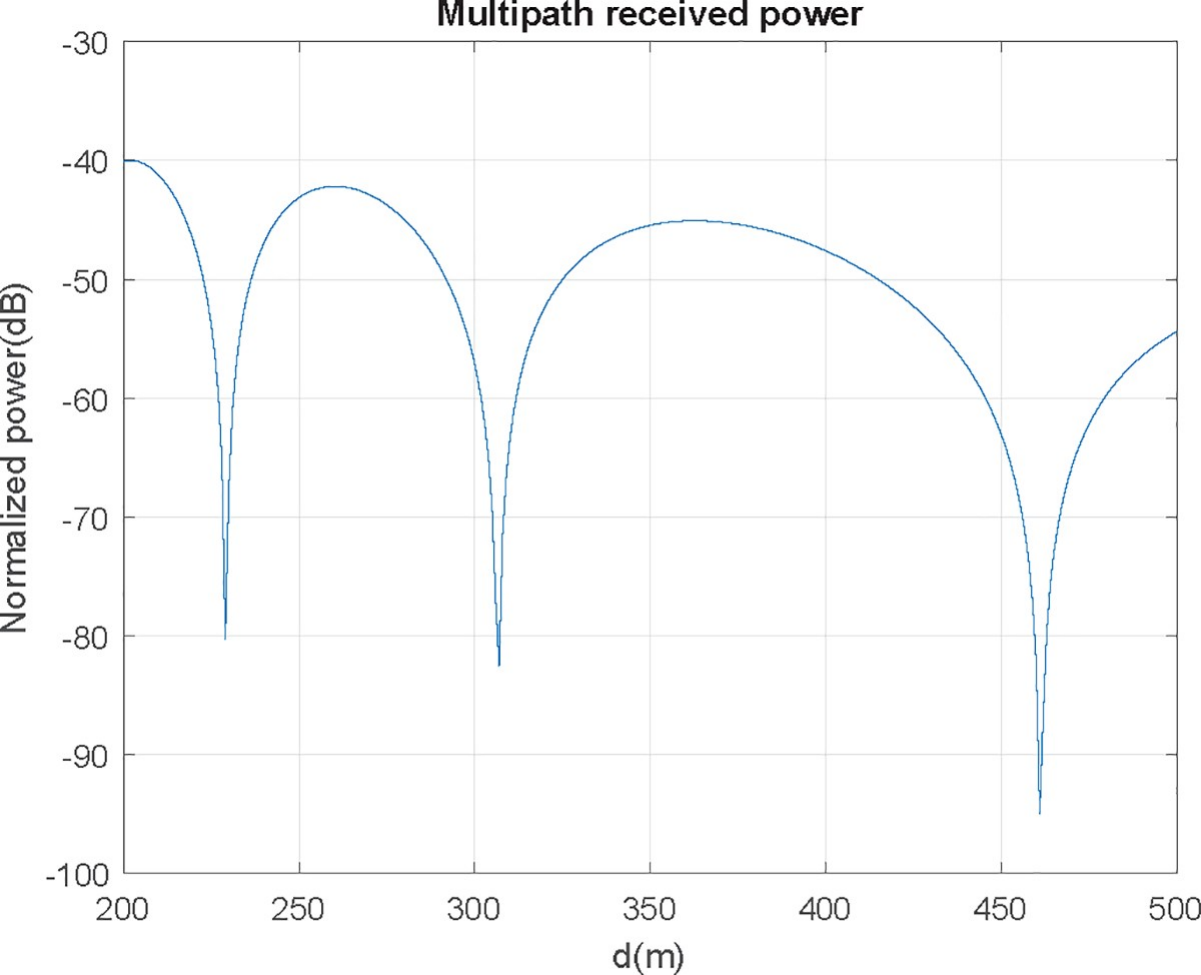
Power received in a multipath scenario for f = 300 MHz, h_UAV_ = h_AUT_ = 21.5 m.

We may observe that using HF under the 220–500 m distance range, the value is always over critical distance (and consequently having no problem). For the rest of frequency bands, multipath lobulation must be considered (as show in section 2.2.3). Using 300 MHz frequency, as depicted in Figs 16 and 17, we may identify two zones: first, a relatively narrow plain zone between 255 and 265 m (where field values keep a gain with respect free space conditions of 4.6 ± 0.2 dB); second, the 350 to 380 m zone (1.6 ± 0.2 dB gain with respect free space conditions). Using 3 GHz there are several chances, where the 445 to 455 m zone shows better attenuation (-6.5 ± 1.25 dB). Finally, for 12 GHz we find again several possibilities, where the 469 to 471 m zone has -7.25 ± 0.75 dB attenuation. As frequency increases lobulations are narrower and, consequently, in the same distance range, the signal will vary more at high frequencies than at lower.

First Fresnel’s ellipsoid radius (*R*_1_) for each frequency was calculated in section 2.2.3 using (5), assuming *h*_*UAV*_ = *h*_*AUT*_. Table 4 shows *R*_1_ against distance and frequency. Only when *h*_*UAV*_>*R*_1_, will Fresnel’s ellipsoid be over the sea, consequently not affecting communication. As seen in section 2.2.2, the minimum flight level lowers as frequency increases, which adds difficulty in measuring HF and VHF antennas. Since UAV’s flight level is *h*_*UAV*_ = 21.5 m, UHF and SHF represent no issue. Using VHF, Fresnel’s ellipsoid will be interfered flying at 500 m distance (and not at 220 m). In HF, it is ineludible invading the ellipsoid flying so low, so flight level needs to be increased.

**Table 4:** First Fresnel’s ellipsoid height for h_UAV_ = h_AUT_ = 21.5 m.

	*h* (m)	
	*d* = 220 m	*d* = 500 m
Coms. HF (1.5–30 MHz)	105–23.5	158–35.5
Coms. VHF (30–300 MHz)	23.5–7.5	35.5–11.2
Coms. UHF (300–3000 MHz)	7.5–2.4	11.2–3.5
Coms. radar SHF (3–12 GHz)	2.4–1.2	3.5–1.8

Table 5 summarizes distance and flight levels for the frequency bands mentioned. Assuming multipath effect for VHF, UHF, and SHF, radiation pattern margin errors for these bands is also considered, assuming the uncertainty due to this issue:

**Table 5:** Radius, flight level and measurement uncertainty for F-100’s class Spanish Navy frigates’ antenna.

	*R* (m)	*e* (m)	*h* (m)
Coms. HF (1.5–30 MHz)	220–500	≈0	>160
Coms. VHF (30–300 MHz)	350-380/255-265	±0.2	>35
Coms. UHF (300–3000 MHz)	255-265/445-455	±1.25	21.5
Coms. & radar SHF (3–12 GHz)	445-455/469-471	±0.75	21.5

### Circle flight path tests

The following simulations provide a verification for the behavior of the parameters shown in previous sections. Every test consists of a short approach flight and the circular path flight, as shown in section 2.3. In all of them, the UAV is oriented in such a way that the receiving antenna (PA) always points to the transmitting antenna (AUT).

#### Polygonal approximation to circle flight

**Fig 18:**
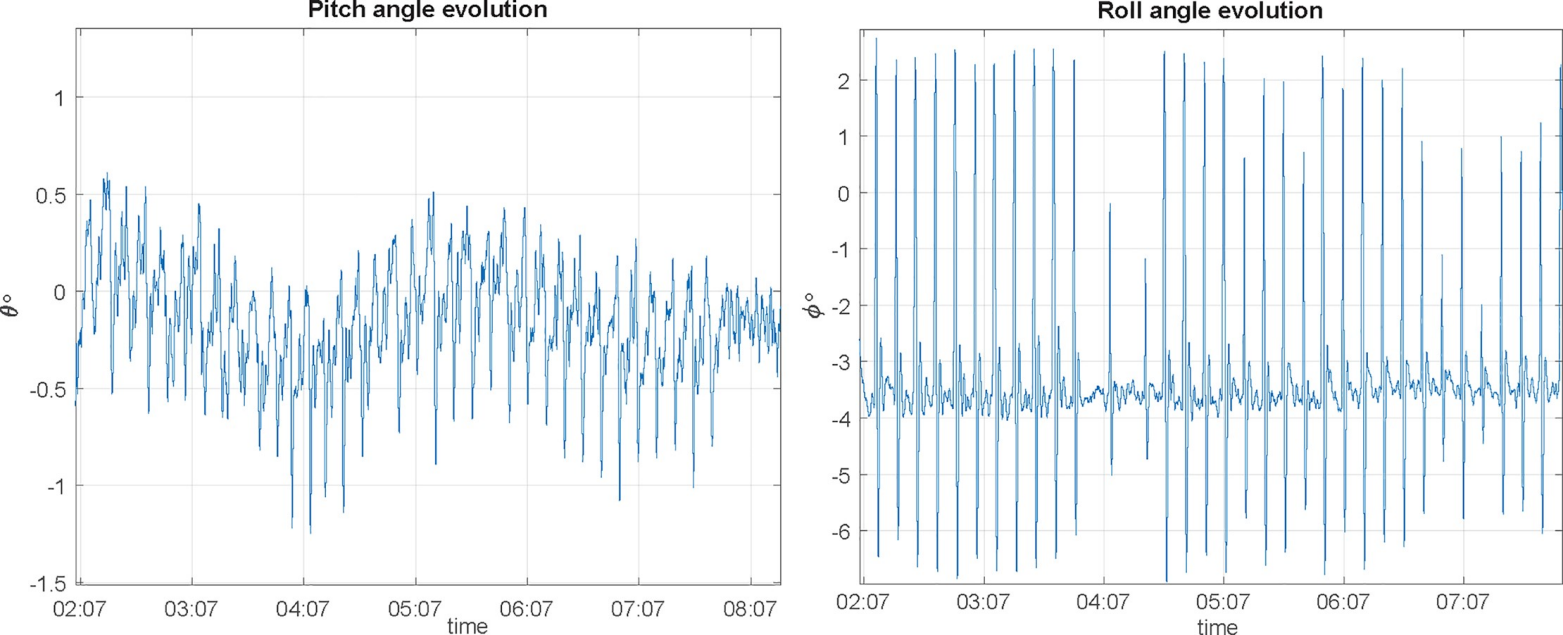
Pitch and roll angle evolution for a 10° polygonal flight at v = 1 m/s, radius 50 m.

As a first test, a 50 m radius flight with a 10° resolution (36 vertex or waypoints) and 1 m/s ground speed (as described in section 2.3.1) is performed. Pitch angle during circle flight has an angular variation of *Δθ* = 1.2° with a 0.8° maximum and as for roll values, angular variation is *Δϕ* = 8° with a maximum value of 6°, as shown in Fig 18, where UAV suffers an abrupt roll variation since it needs to lower its speed to reach the waypoint properly and then accelerates to go for the next vertex.

For the second test, flight speed will increase to 2 m/s. Pitch angle during this flight has an angular variation of *Δθ* = 1.5° with a 1° maximum; as for roll values, angular variation is *Δϕ* = 3° with a maximum value of 11°. As a conclusion, an increase on flight speed adversely affects roll, but almost has no impact on pitch.

A third test is performed to analyze the impact of changing the number of waypoints: a 5° resolution (72 vertex polygon) is used, for a flight speed of 1 m/s. In this case, pitch angle during this test has an angular variation of *Δθ* = 1.1° with a 0.7° maximum and for roll values, angular variation is *Δϕ* = 8° with a maximum value of 6°. This last result shows that an increase on the number of vertexes does not affect measurement, since pitch and roll angles keep constant.

The fourth test is performed changing radius flight to 100 m, with a 10° resolution, and flight speed of 1 m/s. For this case, an angular variation of *Δθ* = 1.2° with a 0.6° maximum is found; as for roll values, angular variation is *Δϕ* = 8° with a maximum value of 6°. Compared to previous values, we may find almost no difference due to the change of radius flight. For 100 m radius, there are lesser UAV’s roll peaks; the reason is because UAV’s navigation system performs less acceleration/deceleration loops while arriving/departing every waypoint to focus to the next step. In conclusion, using greater radius flights is helping the accuracy of the measurement since UAV almost makes no waypoint stops, smoothing flight path.

#### Circle path by flight following a trajectory flight

UAV will follow a circle path keeping flight speed constant, adjusting course constantly so that it keeps tangential to its trajectory. Its navigation system will control off-track error (*d*), adjusting lateral speed. *V*_*x*_ (*x*-axis component) will be constant and will match fixed ground speed; *V*_*y*_ (*y*-axis component) will be proportional to the error (to the distance between actual UAV’s location and the circle trajectory we want to describe). UAV is under “speed mode” (receiving just speed and course commands); for each iteration V_y_ will be calculated to keep UAV’s tangential to its trajectory.

**Fig 19:**
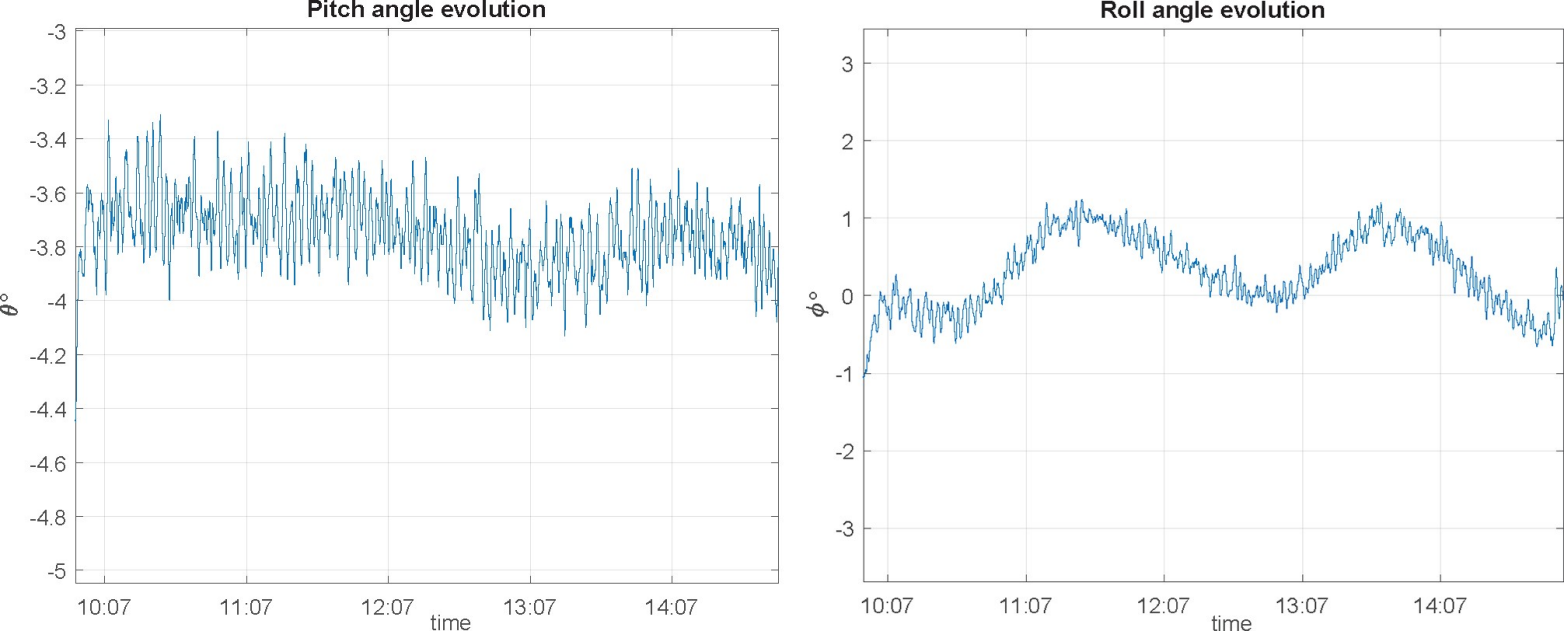
Pitch and roll angle evolution for a trajectory guided circle path, at v = 1 m/s, e_vel_ = 0.2 m/s and R = 50 m.

Using this method (as described in section 2.3.2), a first test is performed with 50 m flight radius, 1 m/s ground speed and a 0.2 m/s error tolerance; we obtain the pitch and roll angles depicted in Fig 19:

For this case, pitch angular variation of *Δθ* = 0.5° with a 4° maximum is found and for roll values, angular variation is *Δϕ* = 1.7° with a maximum value of 1°. Compared to the ones obtained under the same conditions in polygonal approximation, angle variations are better in this case.

A second test is performed using the same flight radius (50 m), increasing ground speed up to 3 m/s. In this case, pitch angular variation of *Δθ* = 0.8° with a 11.5° maximum is found and for roll values, angular variation is *Δϕ* = 4° with a maximum value of 4°. The conclusion is that, since those values are greater than the ones at 1 m/s, speed makes errors increase (as was supposed).

Finally, the last test seeks to demonstrate the effect of using a 0.05 m/s max error speed. Pitch angular variation is *Δθ* = 0.7° now with a 4.2° maximum; with regards roll values, angular variation is *Δϕ* = 1.7° with a maximum value of 1°. No better performance is achieved using a more restrictive control law to speed error.

#### Polarization decoupling effect

**Fig 20:**
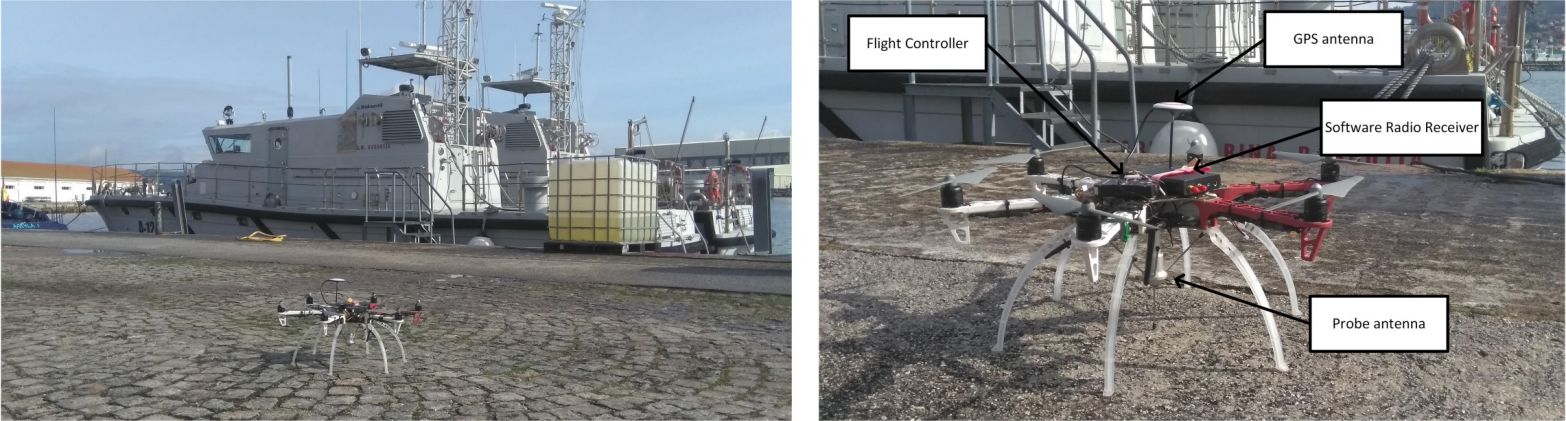
UAV carrying the proposed measurement system: Vertical antenna and software radio receiver.

To check the effect of polarization decoupling while measuring radiation pattern, again a short approach flight will be performed, followed by a circle trajectory flight. Transmission antenna will be a linear polarized vertical monopole located at the center of the circle (reference system’s origin), aligned with *z*-axis. On the UAV receiving antenna is a monopole over an artificial ground plane (similar to the one shown in Fig 20) that will also generate a vertical polarized wave aligned with its own body *z* axis.

**Fig 21:**
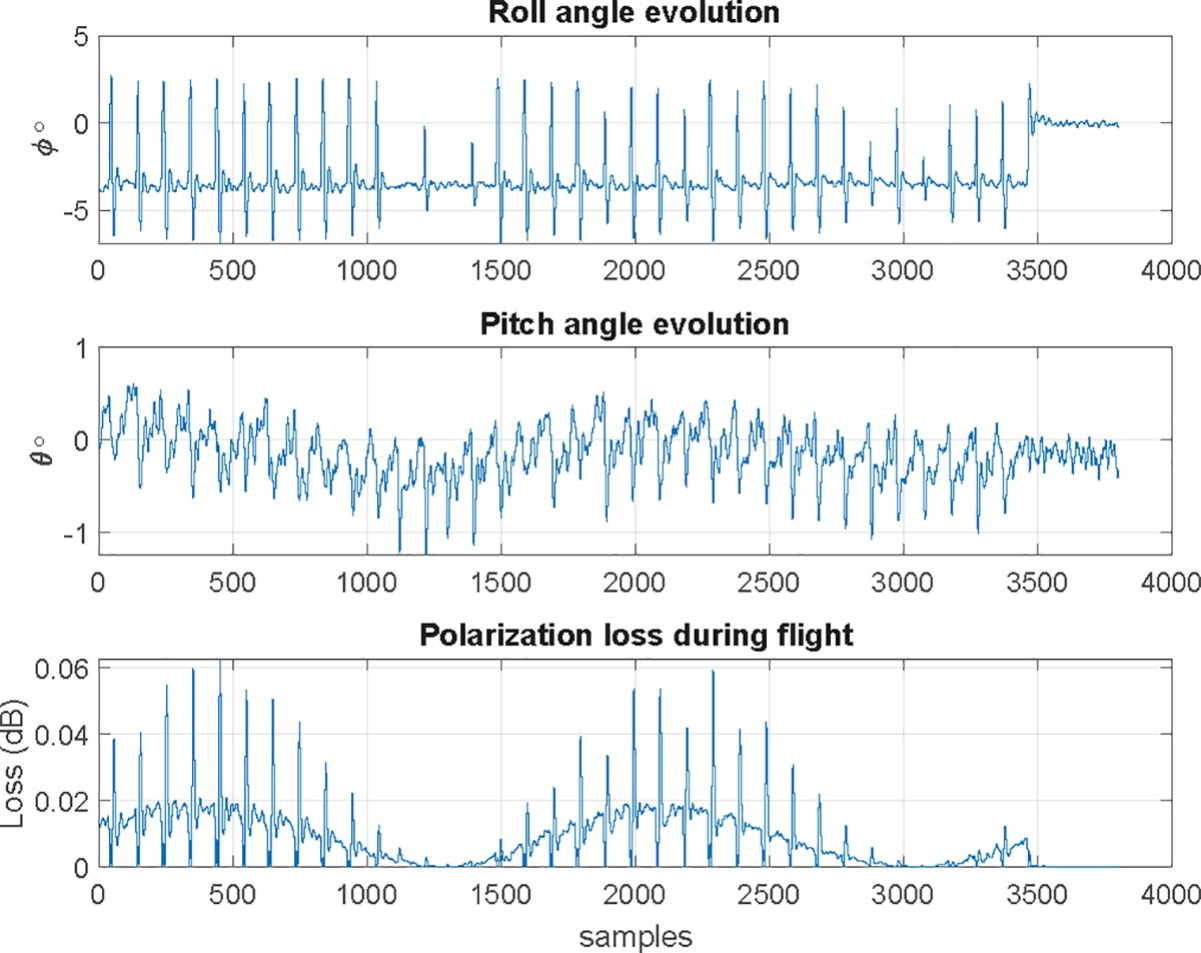
Polarization decoupling losses vs time for a polygonal flight under 10° resolution, 1 m/s ground speed and R = 50 m.

First test is for a polygonal flight under 10° resolution (36 waypoints), 1 m/s ground speed and *R* = 50 m, as shown Fig 21. The maximum loss for this reason is *l*_*pol*_ = 0.063 dB:

The second test is for a polygonal flight 10° resolution (36 waypoints), 2 m/s ground speed and *R* = 50 m, dropping a *l*_*pol*_ = 0.181 dB; it corresponds to a max roll angle *ϕ* = 11.6°, greater than the one obtained for *v* = 1 m/s. The result is as expected because, having increased speed, roll suffers even bigger oscillations than previous case whenever the UAV reaches and departs every waypoint. The third test is for circle flight following a trajectory, characterized by a maximum pitch angle of *ϕ* = 4.13°. For the case of 3 m/s ground speed and a maximum speed error of 0.2 m/s, losses are *l*_*pol*_ = 0.174 dB, corresponding to a maximum pitch angle of *ϕ* = 11.9°. Comparing with previous results, loss and pitch angle have been increased notably. Finally, last test is performed for a 1 m/s ground speed, maximum speed error of 0.05 m/s; for this case, losses are *l*_*pol*_ = 0.022 dB, corresponding to a maximum pitch angle of *ϕ* = 4.4°, almost identical to the ones obtained under *e*_*vel*_ = 0.05 m/s

## Conclusions

In this work, a procedure for the obtention of onboard antenna’s radiation pattern is developed. In this procedure, the ship stays static at the sea transmitting with its AUT while a UAV takes a circular path around the vessel, measuring the received power along that track. After presenting the theoretical analysis of the factors affecting the antenna’s radiation pattern measurement, UAV’s flight envelope factors were also studied to define a reasonable flight volume–ship’s main dimensions, safety distance, far field distance, Fresnel’s distance, and multipath distance. As a result of the analysis and tests utilizing F-100 class Spanish Navy frigate, the following conclusions arise:

- Distances:
  ○ The maximum distance due to optical distance is not a limiting value: it is always beyond standard UAV’s range for this purpose, approximately 120 km.
  ○ The maximum radius (the maximum measurement distance) is conditioned by legislation restrictions (not technical) to 500 m of visual sight from the UAV.
  ○ Fraunhofer’s distance is not limiting either since in the worst-case scenario (for SHF band), the minimum measurement distance is under overall length of most vessels.
    ○ Issues specific to HF are different than the ones that SHF suffers from. Multipath is irrelevant for some frequency bands such as HF and low VHF. At these frequencies, since the critical distance is quite close to the ship, no lobulation exists on the received power. In other bands such as high VHF or UHF, the lobulation is remarkable and must be taken into account, requiring approaches like multipath crown as shown on section 2.2.3 to find a suitable flying zone. Multipath effect at SHF band is always present, specially at a few hundred meters from the ship and must be studied precisely.
  ○ First Fresnel’s ellipsoid need not to be taken care of for UAV’s flight levels that are similar to the ship’s in UHF and SHF bands. For the HF band, it is necessary to increase UAV’s flight level to avoid the ellipsoid. VHF needs a specific case study.
- Circular path strategies:
  ○ Using polygonal approximation circular paths at high ground speed and/or low turning radius, makes the UAV to roll excessively (up to 8°) at waypoint arrival/departure; this affects negatively measurement since it depolarizes transmitter and receiving antennas.
  ○ Path following method has performed better that polygonal approximation, reducing roll up to 1°.
  ○ Ground speed has a negative effect on the path following method, so a reduced speed is advisable (aligned as well to keep UAV’s autonomy)
  ○ Using restrictive speed following error values does not benefit roll and pitch angles.
- Vertical linear antenna’s polarization decoupling:
  ○ Polarization decoupling must be considered, but it is not critical.
  ○ Under small pitch and roll angle flights, losses are almost negligible, within measurement uncertainty. Only 25° pitch-roll angles generate decoupling losses over 1 dB.
  ○ Under polygonal flight, pitch and roll resulted in polarization decoupling with a *l*_*pol*_ = 0.2 dB maximum value, due to the abrupt pitch the UAV experiments at every waypoint.
  ○ Under path following flight, losses due to polarization decoupling are one order of magnitude (*l*_*pol*_ = 0.02 dB) lower than the ones obtained for polygonal flights, because of the absence of waypoints.

Finally, it is important to remind that flying the UAV at the same height as the AUT is a key fact to ease the analysis of the other parameters.
